# Case of Hydrophobia Successfully Treated with Drachm Doses of Calomel

**Published:** 1860-02

**Authors:** John E. H. Ligget

**Affiliations:** Middleburg, Carroll County, Md.


### ECLECTIC DEPARTMENT,

### AND SPIRIT OF THE MEDICAL PERIODICAL PRESS.

---

### *Case of Hydrophobia successfully treated with Drachm Doses of Calomel.*

By JOHN E. H. LIGGET, M. D., of Middleburg, Carroll County, Md.

ON Monday, the 16th July, 1851, I was requested by George Mearing, Esq., of Bruceville, in this county, to visit his colored girl, Maria, aged about 20 years, whom he supposed to be laboring under the above disease. On my way to Mr. M.'s he gave me the following history of the case: Some sixteen or eighteen days previously, this girl, with his little son, eight or nine years of age, was in the yard teasing a young dog that had been unusually dull and morose for a day or two. Whilst holding her naked foot towards him, the dog snapped her in the great toe, and immediately sprang at the child, whom he seized by the arm. The girl ran at once into the house with the child, whose cries quickly alarmed the family. Upon removing the clothing from his arm, the indentations of the dog's teeth were distinctly visible, but the skin was unbroken; and as the girl said nothing of the dog having snapped her, Mr. M.'s fears were quieted. He at once had the dog chained in an out-house, where, in two or three days, he *died* with all the symptoms of *rabies canina* in its most virulent form. Some three days before I was called, Maria complained of pain in the great toe, extending up the limb towards the body; at the same time, from being a very lively girl, she became dull, moody, taciturn, and irritable. Upon being closely pressed by her master, she confessed that the dog had seized her by the toe, and that one of the tusks had penetrated *between the nail and the flesh*, and had drawn blood. Becoming alarmed, Mr. M. went to Littlestown, Pa., to procure a nostrum (prepared by a noted empiric), which enjoys much celebrity as a *prophylactic* in this disease. Upon procuring the article he was told by the "doctor" that, if the disease was near development, she might, whilst taking the medicine, have *one or two* "*fits*," which need not alarm him, as it would indicate that the remedy was producing a "proper effect." He must persevere, and she would soon be relieved of all unpleasant symptoms. Upon returning home he found the girl worse, and she now complained of pain in the epigastrium, with slight stiffness of the muscles of the neck. He gave the medicine according to directions, and sure enough, after several hours, she had a "*fit;*" after some time another, and again another. He, however, persevered until the following morning, when the medicine had all been taken, and the spasms were increasing, frightfully, in frequency and violence. He then called on me for assistance. I found her condition to be as follows: Her mind is clear, and she is conscious of the approach of the paroxysms, of which she usually gives notice. Countenance anxious and despairing; pain in the epigastrium, radiating towards the spine. Stiffness of cervical muscles increased. Urgent thirst, with *inability to swallow fluids*, which are immediately ejected from the mouth with great force. The tongue is white. Pulse 90, and rather tense. Respiration natural, except during the paroxysms, when it is hurried and laborious. There is *increased salivary secretion*, and she occasionally expectorates, with violence, small quantities of viscid mucus, which appears to be thrown from the fauces. The convulsive paroxysms are frequent, and can at any time be excited by touching her, by a current of air, or by the sight of water or other fluids.

I told her master that the disease was undoubtedly hydrophobia; that it had uniformly proved fatal under all known systems of treatment, and that as I proposed to pursue a course he might deem hazardous, I should prefer, before commencing it, to have my *diagnosis* fortified by the opinion of another physician. After requesting that Dr. Swope, of Taneytown, might be sent for, I scarified and cauterized the toe, directed counter-irritants to the spinal column, and left her.

5 o'clock P. M. Dr. Samuel Swope saw the case with me. We found the paroxysms still increasing. Morbid sensibility of surface excessive. Thirst so greatly increased that she now calls constantly for water, the sight of which excites great horror and immediate spasm. In the intervals, complains of pain in the head. Intellect still clear. Pulse 105, tense. Heat of surface somewhat increased. After a careful examination of the case, Dr. Swope concurred in my diagnosis. He also assented to the plan of treatment I proposed, though without any hope of averting the fatal result he anticipated. She was now bled to the amount of ℥ xxxvj, and ordered hydrarg. chlor. mit. ʒ j, to be repeated every *four* hours if the symptoms remain unabated. If the spasms decline in frequency and violence the intervals to be lengthened to *six* or *eight* hours.

17*th*, 8 o'clock A. M. After the exhibition of the calomel last evening she had *one* spasm, after which the spasms ceased until two o'clock this morning, when they returned with much violence. She then took ʒ j hydrarg. chlor. mit., and had an enema administered (which produced but slight effect), after which the spasms again ceased. She is now, 8 o'clock A. M., lying quiet, though in other respects her symptoms are nearly as they were yesterday evening. Ordered a drachm of calomel, to be followed by an enema of ol. terebinth. Thirst to be quenched with spoonfuls of *crushed ice*, which she swallows with difficulty, her eyes being closed to avoid the sight of it. She is to be kept perfectly quiet in a darkened room, and all causes of irritation carefully avoided.

5 o'clock P. M. The bowels have been moved moderately, dejections nearly natural. She has been free from the convulsive paroxysms until within the last hour, when they returned, but with less violence. ℞ — Hydrarg. chlor. mit. ʒ j, to be repeated in eight hours should there be any return of spasm. Continue ice *ad libitum*.

18*th*. Could not visit the patient this morning, but learned from Mr. M. that she had rested quietly since the last dose of calomel. Directed ice to be continued, and the bowels to be moved by enema, ol. terebinth.

4 o'clock P. M. No return of convulsion since last report. Bowels have been freely moved by enemata; dejections green. Pulse 108, small. Tongue heavily coated. Some heat of surface. Complains of burning pain in epigastrium with tenderness on pressure. Thirst still considerable, but dread of fluids and inability to swallow them continue. Symptoms of approaching *ptyalism.* ℞—Epispast. to epigastrium, and continue ice.

*19th*, 3 o'clock A. M. Patient had a slight spasm yesterday evening shortly after I left, which recurred in half an hour, when her master gave her hydrarg. chlor. mit. ʒ ss, which quieted her till two o'clock this morning, when she complained of violent spasmodic pains in the jaw and inferior extremities, when I was sent for. Ordered tr. opii ʒ j, to be repeated if necessary. Repeat enema.

5 o'clock P. M. Rested, from the effects of the opiate, until one o'clock this afternoon, when, complaining of some pain in the jaw, she took tr. opii ʒ ss, which gave her speedy relief. Blister has drawn well, and greatly relieved the burning at the stomach. Mouth getting decidedly sore. She can now begin to swallow fluids, though with difficulty. Asked for food, and took a little corn gruel. As the bowels have not been opened since last evening, give her ol. ricini ℥ j, to be repeated every three or four hours until the bowels are freely moved. Repeat anodyne, should there be any nervous commotion.

*20th*, 2 o'clock P. M. Bowels have been freely moved, dejections dark green. Mouth deeply ulcerated but dry, and ulcers rather *livid* in appearance. Has been easy since last evening, and slept pretty well during the night. Has taken corn gruel several times to-day, and can now swallow fluids without much difficulty. Pulse 106, and weak. Exhaustion considerable. Ordered quinia disulph. gr. iij, with acid. sulph. aromat. dilut. gtt. v, every three hours. Gargle the mouth frequently with infusion of white oak bark and alum sweetened with honey.

*21st*, Evening. Salivary glands are discharged freely. Ulcers have assumed a healthy appearance, and she appears to be decidedly improving. Continued treatment.

*24th.* Has continued to improve since last report. She is now lively and cheerful. Appetite good. Evacuations natural. Mouth healing.

The subsequent treatment consisted in the regular administration of nutritious diet, tonics and laxatives, with an occasional anodyne; and she was discharged cured on the 28th.

*Remarks.*—I related the above case, shortly after its occurrence, to a medical gentleman residing in an adjoining county, whose venerable years and high professional attainments command the respect of all who know him. He expressed the opinion that I was mistaken in my diagnosis, and gave as his reasons—*first*, that the patient was a *female*, and, *secondly*, that the case *did not terminate fatally*. He assumed from the *sex* of the patient that it was a case of that protean disease, hysteria, *simulating* hydrophobia. He frankly added that had the patient been a *male*, or had she *died*, he would have felt himself constrained to acknowledge it to have been a case of rabies. Now, as other gentlemen may be inclined to entertain similar objections, it may be proper to endeavor to dispose of them in the outset. And first: Is it true that poisons—animal, vegetable, or mineral—have their action upon the human organization so modified by *sex* that they will produce in the *female*, diseases which, although apparently identical with those excited in the *male*, are, nevertheless, mere *counterfeits*, less formidable in their nature, and more amenable to the resources of science ? Will the virus of the rattlesnake or viper, or the exhalations from the Pontine marshes, produce in the female affections *similar* in their *phenomena*, yet *differing* in their *nature*, from those produced in the male ? The records of medicine, so far as I am aware, furnish nothing to sustain such an assumption. Why, then, shall the virus of a rabid animal produce, in the female, a disease presenting all the appalling characteristics of *hydrophobia*, but which, nevertheless, we must take it for granted is *only hysteria*, because the patient *is* a female ? Such a doctrine would forever unsettle our diagnosis, not only in hydrophobia, but in *all other forms of nervous disease* in females. For instance, if a woman receives a wound in a tendinous part, which, after a time, is followed by symptoms of *tetanus*, who shall say that it is not, in reality, hysteria assuming that disguise ? More particularly should the patient recover. That, in the case I have reported, the disease was produced by a poison deposited in the wound when inflicted, will, I think, scarcely be doubted.

The *second* objection presupposes that hydrophopia is *essentially*, *and*, *in its very nature*, incurable. To argue that because certain diseases have hitherto resisted the best directed efforts of medical science for their relief, they are, therefore, *necessarily incurable*, is, to say the least of it, illogical. Our want of success may, more rationally, be attributed to ignorance of the pathological conditions existing in this class of diseases, and consequent inability to employ proper therapeutic agencies for their removal. This is, unquestionably, the case in hydrophobia. Then can it be cause of astonishment that in the present state of our knowledge (or rather *ignorance*) of this malady we should be totally at fault in establishing proper indications for its cure ?

The author of this paper had his attention drawn to this subject at an early period. The mysterious nature of the affection, the awful sufferings it entails, and the fearful fatality with which it siezes upon its hapless victims, invested it with an interest that irresistibly led him to seek for all the information he could obtain on the subject. After availing himself, as far as possible, of all the light that had been shed upon the subject by others, he was pained and humiliated to perceive how little had been accomplished in this particular field of pathological science.

All our assumed knowledge of the pathology of this disease is based on vague, unsettled hypothesis. Nothing has been rendered certain.

Man, as a rational being, when foiled in his efforts to unfold the mysteries of nature, is apt, nevertheless, to form certain *opinions* in regard to the matter he is investigating. These he connects into a *theory*, which, however crude and erroneous it may appear to others, *he* will hug to his bosom as TRUE until its fallacies are exposed. The writer of this article, availing himself of the labors of others,—accepting such hypotheses as commend themselves as reasonable, and rejecting others his judgment cannot sanction—has constructed a *theory* of this disease. His poor efforts, humble though they be, and, perhaps, based upon error, cannot retard the progress of knowledge in a field thus far barren of results ; whilst others, possessing superior facilities for investigation, may be stimulated to renewed exertions, from which the most beneficial results may flow.

Briefly, then, the views which have forced themselves on the writer, as being most in conformity with the known phenomena of the disease, are as follows :—

1st. The hydrophobic virus is an *irritant poison* whose action is directed, *primarily* and *directly*, on the great nervous centres, producing a perversion of their action upon the entire organization, and thus, *secondarily and indirectly*, deranging the functions of the other organs of animal life.

2d. This virus, when deposited in a wound, remains for an indefinite period of time, locked up at the seat of injury, harmless and inert, until some *exciting cause* (of the nature of which I shall not hazard even a conjecture) occasions it to be *absorbed into the circulation*, whence it is carried to the brain and spinal cord to initiate its work of suffering and death.

3d. The primary effect of the poison on the cerebro-spinal system is to depress its action. This is succeeded by great exaltation of the sensibility and irritability of the nervous system, which progressively increases until there is a total exhaustion of the vital forces, and death results from asthenia.

4th. The dread of water or other fluids results from the suffering produced by the effort to swallow them—such effort inducing violent spasm of the muscles concerned in deglutition.

5th. The increased flow of saliva appears to be a conservative effort of the vis medicatrix to *eliminate the poison from the system* through the glands engaged in its secretion.

This is a condensed statement of the views I have been led to entertain in relation to this disease. A very few observations in explanation and support of them may be permitted me. That the action of the poison is directed primarily on the brain would appear from the fact that, in the *first* stage of the disease, the functions of the circulatory, respiratory, and digestive systems, seem but slightly, if at all, impaired; whilst dullness and pain of the head, dejection of spirits, increased irritability of temper, restlessness, unusual timidity, which causes the patient to start at every sound, with a general feeling of *malaise*, undoubtedly indicate a morbid condition of the nervous centres. That the symptoms which accompany the *second* stage (and which it is unnecessary to recapitulate) are clearly referable to morbid or perverted *innervation*, is, I presume, unquestionable. The pathological appearances after death, uncertain and inconstant as they are, bear me out in this conclusion.

Many writers upon this disease suppose that the poison is locked up at the seat of injury during the period of incubation. That it produces its morbid effects upon the system through the medium of the nerves with the sentient extremities of which it lies in contact; and they generally concur in the opinion that it is *not absorbed*, because no traces of pain, swelling, or tenderness, exist in the lymphatics or absorbent glands. Now, if the hydrophobic virus produces its effects through the nerves with which it is in contact, how shall we account for the long period of incubation between its deposition in the wound and the appearance of the disease? Would it not be more reasonable to suppose it would *act at once* upon coming in contact with the nervous filaments, especially as from recent laceration their sensibility and excitability would be morbidly increased? The absence of disease in the absorbent system will not militate against the doctrine of absorption if the hypothesis be admitted that the poison is an irritant whose effects are exerted *directly* upon the nervous centres, affecting the other animal functions, *indirectly*, through the perverted action of those centres. Neither will the *appearances* of inflammation, frequently found in various organs after death, prove fatal to the views here advanced. These appearances are, doubtless, deceptive, and are produced, not by *inflammation*, but by simple capillary *congestion*. The brain, pharynx, stomach, and air-passages, are the usual seats of these appearances; yet where. during life, are the symptoms that would indicate acute inflammatory action in these organs? The absence of delirium, hoarseness, cough, and dyspnœa, and of extreme gastric irritability, sufficiently refute the idea that these appearances are due to inflammation. It is more probable that the terrible muscular convulsions to which the patient is subject, by their disturbing effects upon the organs of respiration and circulation, favor the accumulation of blood in the capillaries, and thus give rise to these morbid appearances. But a more powerful argument in favor of the doctrine of absorption may be found in the fact—now generally conceded—that the virus is contained *in the saliva*, through which it is propagated from one animal to another. Now, as all the secretions are derived from the blood, how could this poison be found in the saliva unless first absorbed into the blood?

The positions assumed in my *third* proposition are, apparently, so well sustained by the symptoms of the first and second stages that to enter upon their discussion in this paper would seem to be unnecessary, involving, as it would, much useless repetition. But as differences of opinion may, and doubtless do, exist as to the immediate cause of death, a word or two in that connection may not be inappropriate.

It is contended by some writers that death is occasioned by an arrest of the respiratory processes, or *asphyxia*. That hydrophobia does, sometimes, terminate in this way in consequence of severe and prolonged *spasm of the glottis*, will not be denied. But that such is the *natural* or even *usual* manner in which the fatal result is produced, I am by no means prepared to acknowledge. That the natural tendency of great and prolonged irritation of the nervous centres is to occasion an *exhaustion of their excitability*, and consequent extinction of the functions of animal life over which they preside will, I apprehend, scarcely be controverted. That such prolonged irritation of the nervous centres does exist, in its highest form, in this affection, is abundantly manifested by all the phenomena which attend its progress. Besides, many cases are on record where all muscular commotion had ceased some time anterior to dissolution, and where death was evidently the result of a total exhaustion of the vital forces. I feel bound, therefore, to assume this to be the *natural* and usual mode in which death is produced, unless interrupted by the casualty adverted to.

That the *dread of fluids* is occasioned by the spasm of the muscles concerned in deglutition, which an attempt to swallow them excites, is not a new opinion. It has been held by many of the ablest writers on the disease. But why *fluids* should, preferably, or in a greater degree, excite spasm than solids, I am unable to explain; unless, indeed (in the state of exalted exitability of *all the senses* during the second stage of the disease), the *oscillatory motion* of fluids, or their more *sudden diffusion* throughout the buccal cavity, should prove to be the cause. The solution of this problem, however, I leave to older and wiser heads than my own.

"The increased flow of saliva appears to be a conservative effort of the vis medicatrix to eliminate the poison from the system, through the glands engaged in its secretion."

From the novelty of this proposition (which, so far as I am aware, is exclusively my own), it will naturally be expected that it shall be supported by an elaborate series of arguments tending to sustain its pretensions as a scientific truth. Candor compels me to acknowledge my inability to do this in a manner satisfactory even to myself; and were it not that it is the *keystone* upon which my indications of cure are based, I should hesitate long before committing it, thus unsupported, to the tender mercies of the professional critic.

Yet, however erroneous it may prove to be when tested in the crucible of scientific analysis, with the results of my single successful case in view I shall continue to cherish it *as true* until its fallacies are demonstrated. I am aware that high authorities have denied the existence of *increased* salivary secretion in hydrophobia, because no evidences of *disease in the glands* can be detected. They infer that the frothy fluid which flows from the mouth, or adheres to the lips of patients and animals, is derived from the air-passages; and refer, in support of this view, to the fact that large quantities of a similar fluid are frequently found in the bronchial tubes and air-cells of the lungs after death.

To argue that because a secreting organ may not have manifested signs of *disease*, therefore its secreting power could not have been increased, is illogical and contrary to well-known facts in medical science.

As to the assumption, that the frothy fluid which flows from the mouth is formed in and proceeds from the air-passages, it will, I apprehend, require more evidence than has yet been adduced in its support, to entitle it to much consideration. In all the cases of hydrophobia I have seen upon record, I have yet to observe among the symptoms enumerated, any reference to the *physical signs* indicating accumulations of fluid in the air-passages of the lungs. Surely had such accumulations existed before death, the physical signs denoting them would have been sufficiently prominent to have claimed for them especial attention. But, again, if the frothy fluid be formed in the bronchial tubes, it must, before reaching the mouth, pass through the trachea and larynx. Could it do so without exciting violent paroxysms of spasmodic cough and dyspnœa?

Reasoning, then, from these general deductions, I assume that there *is* increased salivary secretion; that this secretion *contains the virus by which the disease is produced*, and which is separated from the blood by the secretory action of the glands. Hence, I infer that the increased action of the glands is a conservative effort of nature to rid the system of the poison. Now, if these assumptions be correct, what are the plain and palpable indications they suggest for the management of the disease? Clearly, *first*, to reduce, if possible, the extreme excitability of the nervous system, the continuance of which occasions rapid exhaustion of the vital powers, and death; and, *secondly*, to followthe path *pointed out by nature*, and endeavor to aid her in her efforts to expel the poison from the system through the channel she has indicated.

But where are the remedies capable of fulfilling these indications? If we are to rely on the recorded experience of the past, we shall find this a most difficult question to answer. Blood-letting, mercury, the whole class of narcotics and antispasmodics, have in turn been tried, and thrown aside as useless. Indeed, the entire materia medica has been laid under contribution, and almost every article possessing any activity has been resorted to, with the same unvarying results.

Where, then, are we to look for an agent capable of controlling this formidable disease? MERCURY I believe to be *the* remedy for hydrophobia. But to be efficient, it must be carried to the full extent of its *constitutional powers*, by which we shall be enabled to fulfill the *second* indication I have laid down for the treatment of the disease. But the query, "How are we to accomplish the *first* indication?" naturally presents itself; and I answer "With the same remedy—mercury!" In the autumn of 1849, I witnessed the surprisingly prompt effects of calomel, in doses of sixty and eighty grains, in arresting the spasmodic action of the muscles, and checking the vomiting and purging, attending two cases of cholera which occurred in my practice—this, too, after a fair trial had been given to calomel and opium, administered every half hour, in the usual doses, and when the patients were livid and nearly pulseless. From the results in these cases, I was led to suppose that calomel, *in large doses*, might prove as efficient in restoring the equilibrium of the nervous system when disturbed, as it is in equalizing the circulation in some other forms of disease. From the notes of Maria's case, it will be perceived that the convulsive paroxysms were suspended for a period of *eight hours* after the first dose of calomel; and after the third dose nearly *twenty-four hours* elapsed, during which she was entirely exempt from them. It was not, however, until the full constitutional effects of the medicine were obtained that they entirely disappeared.

Since I commenced this paper, my attention has been attracted to a case of hydrophobia, reported in 1811, by a Mr. Tymon, a surgeon in the East Indies, who claims to have cured the patient by *large abstractions of blood*. He says, "I began by bleeding him until scarcely a pulsation could be felt in either arm." But he adds, "Opium was afterwards given, and the patient *salivated with mercury*." In my case, it will be observed, bloodletting was also practiced, though not to the same extent as in the case treated by Mr. Tymon. I resorted to it, however, with no expectation that, of itself, it would exercise the slightest controlling influence over the disease; but simply for the purpose of obviating any tendency there might be to cerebral congestion, and for the further purpose of *promoting the more rapid absorption of the mercury*, by lessening the mass of the circulating fluids; and these, I apprehend, were the only beneficial effects produced by the large abstraction of blood in Mr. Tymon's case.

Insusceptibility to the action of medicines is a marked feature in this disease. Almost incredible quantities of opium and other narcotics have been given, without producing even the slightest degree of narcotism. This fact should be borne in mind in our efforts to bring patients under the constitutional effects of mercury; together with the additional fact that, from the rapidly fatal character of the disease, but little time is given in which to effect this object. By giving calomel in the doses here recommended (if my theory of its action on the nervous system be correct), we not only retard the fatal exhaustion resulting from the extreme excitability of the nervous centres, and thus gain time, but we rapidly saturate the system with the medicine, and obtain its *specific effects* in a much shorter period than by the ordinary mode of administering it.

I have thus, as concisely as possible, stated the views in relation to hydrophobia, that have appeared to me to be most in conformity with the known phenomena of the disease, together with the indications of cure they suggest. That the whole theory here advocated may not be founded in error, I am unprepared to assert. If so, it will share the fate of all that have preceded it. But as my object in laying it before the profession is simply to invite renewed attention to a disease that, from its supposed incurability, has, I humbly conceive, been too much neglected, I am willing it shall run the gauntlet of experiment and criticism, in the hope that some beneficial results may be attained.

The science of medicine, within a few years past, has, by the aid of analytical chemistry and the microscope, progressed with a rapidity unparalleled in any former age of the world. Why may not these powerful aids be invoked in the investigation of this disease? May not the subtle poison which produces it be isolated, and its sensible and chemical properties demonstrated? And might not the microscope detect lesions of structure that are undiscoverable by the naked eye?

Gentlemen who enjoy facilities for investigation not possessed by the humble country practitioner, would do well to turn their attention to this subject. It is a goal worthy their highest ambition; for he who shall strip hydrophobia of its terrors, by demonstrating its pathology and laying down a rational system of treatment for its cure, will carve out for himself a lofty niche in the Temple of Science, and place his name among the benefactors of his race. I would rather be *that man* than to sway the sceptre of an empire.—*American Jour. of Med. Scienee.*

